# Relationship Between Obesity and Intellectual/Developmental Disability in an Ohio Telepsychiatry Clinic: A Retrospective Review

**DOI:** 10.1007/s10803-024-06432-0

**Published:** 2024-06-22

**Authors:** Sana Shameem, Megan Luft, Michael Harrington, Ramzi W. Nahhas, Michael Hatesohl, Julie Gentile, Danielle Gainer

**Affiliations:** 1 Boonshoft School of Medicine, Wright State University, Dayton, OH USA; 2 Present Address: Department of Psychiatry, Wright State University, Dayton, OH USA; 3 Department of Population and Public Health Sciences, Wright State University, Dayton, OH USA

**Keywords:** Intellectual/developmental disorder, Intellectual disability, Autism spectrum disorder, Obesity, Telepsychiatry, Community psychiatry

## Abstract

Co-occurring intellectual/developmental disability (IDD) and overweight/obesity (OW/OB) is an important consideration of IDD psychiatric care. The relationship between OW/OB and comorbid diagnoses of Autism Spectrum Disorder (ASD) and/or IDD remains inadequately described in existing literature. The purpose of this study is to explore these co-occurring diagnoses. Improved understanding of associated comorbidities can guide clinicians toward interventions to minimize complications associated with OW/OB. We conducted a retrospective review of adult patients of a telepsychiatry clinic with IDD or ASD defined by DSM-5. ICD-10 diagnosis of IDD or ASD, demographics, BMI, comorbidities, and current medications were recorded. Binary logistic regression was used to estimate associations between each predictor and the outcomes overweight (body mass index (BMI) ≥ 25 kg/m^2^) and obesity (BMI ≥ 30 kg/m^2^). Prevalence of obesity in these 412 adults was 52.4% (95% CI 47.5, 57.3). There was a significant inverse relationship between IDD severity and the odds of each outcome (p < .001). 80.3% of patients were being actively treated with an antidepressant. Patients taking an antidepressant had twice the odds of obesity (adjusted OR 2.03, 95% CI 1.23, 3.41, p = .006). These findings provide a sense of urgency for prevention of OW/OB and its associated medical sequelae. Prevalence of obesity was higher in this sample compared to the general population. The inverse relationship between IDD severity and OW/OB warrants further research examining age, caregiver involvement, and access to care as potential modifiers.

The prevalence of overweight/obesity (OW/OB) in the United States continues to rise (Wang et al., 2020). The World Health Organization (WHO) estimates that the worldwide prevalence of obesity nearly tripled between 1975 and 2016. OW/OB for the general population has known associations with metabolic syndromes and these associations become more complex when considering patients with intellectual/developmental disability (IDD). That is, those with intellectual disability, Autism spectrum disorders, or both often have complex medical and psychiatric histories that can overlap or be mistaken as one another. It has become increasingly important to examine how specific populations may be impacted by OW/OB. Prior literature shows that patients with IDD appear to be at increased risk of OW/OB compared to the general population (Ranjan et al., 2018). Importantly, obesity is considered one of the most preventable secondary conditions among people with disabilities (Rimmer et al., 2011). However, providers, caregivers and other individuals that support patients with IDD would benefit from the elucidation of which sub-groups within IDD populations may be at a higher risk for OW/OB. This can heighten awareness of metabolic syndromes and their associated sequelae. This holds significance for patients, clinicians and caretakers on an individual health and societal level.

Several studies have already explored the relationship and potential correlates between intellectual disability (ID) and OW/OB. In a cross-sectional observational study conducted in Ireland, Ryan et al. (2021) investigated the prevalence of OW/OB and chronic health conditions in older adults (age ≥ 40) with ID. They found a higher prevalence of OW/OB in women than in men. In another cross-sectional study conducted in the Netherlands, including 980 patients with borderline intellectual functioning to profound ID, de Winter et al. (2012) found that older, female patients were at increased risk of OW/OB. Patients with Trisomy 21 or active treatment with antipsychotics were also at increased risk. Hsieh et al. (2014) noted that individuals who were taking medications that cause weight gain were more likely to be obese. They also found a higher prevalence of OW/OB in women compared to men, similar to previous studies. Ptomey et al. (2020) conducted a study examining children and adults with IDD and found that adults with Down syndrome, ASD, and other ID have a higher prevalence of OW/OB compared to the general population. The authors found that adults with Trisomy 21 had the highest rate of OW/OB, while adults with ASD and adults with other IDD had rates comparable to each other but still lower than seen in adults with Trisomy 21.

When considering the relationship between obesity and IDD, it is important to consider social determinants, including socioeconomic status and level of care or independence in the community. Current research related to these factors is limited. Few studies have researched the nuanced living environments and lifestyle in this patient population, often with various limitations regarding supported residential setting, work, diet, activity, and socialization. In a cross-sectional observational study conducted in Ireland, there was a higher prevalence of OW/OB in those who lived independently or with family rather than in a community group home (Ryan et al., 2021). In another cross-sectional study, participants with less severe ID, more independent functioning, and less physical activity had increased risk of OW/OB (de Winter et al., 2012). The overall wellness and health outcomes of those with both IDD and OW/OB are also related to other comorbidities those patients experience. Adults with IDD face comorbidities with a widespread impact on several systems—primarily cardiovascular and endocrine systems, metabolism, and mental health.

This study examines the relationship between obesity and IDD in the patient clinical sample of Ohio’s Telepsychiatry Projet at Access Ohio Mental Health Center of Excellence. This telepsychiatry clinic treats adult patients with varying degrees of IDD and co-occurring psychiatric conditions, socioeconomic backgrounds, and areas of living throughout the state of Ohio, serving 85 of 88 of Ohio’s counties.

The primary aim of this study is to estimate the prevalence of OW/OB in the clinical sample of Ohio’s Telepsychiatry Project. The authors of this study hypothesized that obesity would be more prevalent in this specific sample than in the United States or Ohio. The prevalence of obesity in the United States is 41.9% (95% CI 39.4%, 44.3%) as of 2020 (Stierman et al., 2021) and in Ohio is 37.8% (95% CI 36.6%, 39.0%) as of 2021 (Centers for Disease Control and Prevention, 2021) The secondary aim is an exploratory analysis to identify factors associated with being overweight or obese, for example, having ASD only (no intellectual disability) compared to co-occurring ASD + ID (intellectual disability), as well as ID severity, medications, and patient demographics. In brief, the authors sought to answer: (1) what is the prevalence of OW/OB in the given sample? and (2) what are other factors (demographics, co-occurring diagnoses, etc.) that are associated with OW/OB status?

## Methods

### Design and Setting

Data were collected by means of a retrospective chart review of 605 Ohio Telepsychiatry Project/Access Ohio patients. Once inclusion criteria were confirmed, study data were collected and managed using REDCap electronic data capture tools hosted at Wright State University. REDCap (Research Electronic Data Capture) is a secure, web-based software platform designed to support data capture for research studies, providing (1) an intuitive interface for validated data capture; (2) audit trails for tracking data manipulation and export procedures; (3) automated export procedures for seamless data downloads to common statistical packages; and (4) procedures for data integration and interoperability with external sources (Harris et al., 2009, 2019).

### Participants

Participants were adult patients seen in the Ohio Telepsychiatry Project/Access Ohio clinic. This outpatient telepsychiatry clinic treats adults with varying degrees of IDD (that is, those with ID, ASD, or both) and co-occurring psychiatric conditions. Patients live throughout the state of Ohio, with the Ohio Telepsychiatry Project/Access Ohio clinic serving 85 of 88 of Ohio’s counties.

The sample was constructed by running a de-identified report through the electronic health record (EHR) of patients with seen at Access Ohio. Inclusion criteria were age 18 years old or older, IDD diagnosis (diagnosis of ID or ASD as defined by DSM-5) and seen at least once by a psychiatric physician from January 1st, 2021, through December 31st, 2021. ID diagnoses were further stratified by severity based on ICD-10 code: mild, moderate, severe, profound, other/unspecified. Data were extracted and entered into REDCap during 2022. Of the 605 total charts reviewed, 412 charts were included in this study. One hundred twenty-three charts were excluded due to the most recent encounter with a physician not occurring in 2021. Fifty-three additional charts were excluded due to the lack of ID or ASD diagnosis. Finally, 17 charts were excluded due to a missing BMI value.

### Measures

Variables included in analyses were ID/ASD status, ID severity, age, BMI, sex assigned at birth, minority status, genetic disorders by category, psychiatric and/or medical comorbidities by category, prescription medications by category, and community need index (CNI). ID was categorized as mild, moderate, severe, profound, other, and unspecified. Obesity status was categorized as: 25 ≤ BMI < 30 kg/m^2^ being overweight, 30 ≤ BMI < 35 kg/m^2^ being obese class I, 35 ≤ BMI < 40 kg/m^2^ being obese class II, and BMI ≥ 40 kg/m^2^ being obese class III. Obesity prevalence in this sample (obese class I or greater) was compared to the 2020 estimate of prevalence in the United States, 41.9% (95% CI 39.4%, 44.3%) (Stierman et al., 2021) and to the prevalence in Ohio as of 2021 which is 37.8% (95% CI 36.6%, 39.0%) (Centers for Disease Control and Prevention, 2021).

CNI is a zip code-based index where higher scores indicate greater barriers to health care. This tool aggregates income, culture/language, education, housing status, and insurance coverage and applies them to every zip code in the United States (Roth & Barsi, 2005) In this framework, there is a scale from 1.0 to 5.0, where 1.0 indicates a zip code with the least need and 5.0 indicates a zip code with the most need. CNI was selected for this study to adjust for its potential confounding of the association between ID severity and OW/OB. Descriptive statistics were computed for each analysis variable (frequency and proportion for categorical variables, mean and standard deviation for continuous variables).

### Procedure

If available, patient sex, age, race/ethnicity, and zip code were automatically imported into REDCap from the EHR’s generated query report. ICD-10 diagnosis of ID and/or ASD, BMI, comorbidities, and current prescription medications with associated drug class were manually extracted from the EHR and entered into REDCap by research team members. Data were verified by two different people to ensure accuracy.

### Analysis

For the primary analysis, the following prevalences and 95% confidence intervals were estimated (including a continuity correction): (1) Prevalence of BMI in the intervals 25 ≤ BMI < 30 (overweight), 30 ≤ BMI < 35 (obese class I), 35 ≤ BMI < 40 (obese class II), and BMI ≥ 40 (obese class III); (2) Prevalence of BMI in the intervals BMI ≥ 25 (overweight+), BMI ≥ 30 (obese), BMI ≥ 35 (obese class II–III); and (3) Prevalence of overweight+, obesity, and obese class II–III by ID severity (None, Other/Unspecified, Mild, Moderate, Severe, Profound) (*Defining Adult Overweight & *Obesity, 2022). The purpose of this analysis is to determine the prevalence of OW/OB in this clinical sample.

For the secondary analysis, binary logistic regression was used to estimate unadjusted and adjusted odds ratios and 95% confidence intervals and test associations between each predictor and the outcomes overweight+ and obesity. Adjusted models were adjusted for age, sex, and CNI. We did not include all the predictors together in one multivariable model as doing so would result in overfitting. Finally, we investigated the possibility that age acts as a modifier of the association between ID severity and OW/OB by including an ID severity × age interaction in each model. The purpose of these secondary analyses was to investigate potential associations between odds of OW/OB and ID status, ASD status, ID + ASD co-occurring status, ID severity, medications prescribed, and co-occurring medical conditions.

### Ethical Considerations

While formal informed consent was exempt for this study from Wright State University’s institutional review board, it is essential to maintain confidentiality for all participants. This was in part made possible by utilizing REDCap for data capture. Efforts to ensure accuracy of data included one person entering data into REDCap with another person verifying thereafter. Care was taken in ensuring that data extracted from private electronic medical records was used solely for this research. Care should also be taken in presenting findings associated with these vulnerable populations to not reinforce stigma and negative stereotypes. The authors aimed to provide data that can benefit this population’s overall wellbeing and health.

## Results

**Fig. 1:**
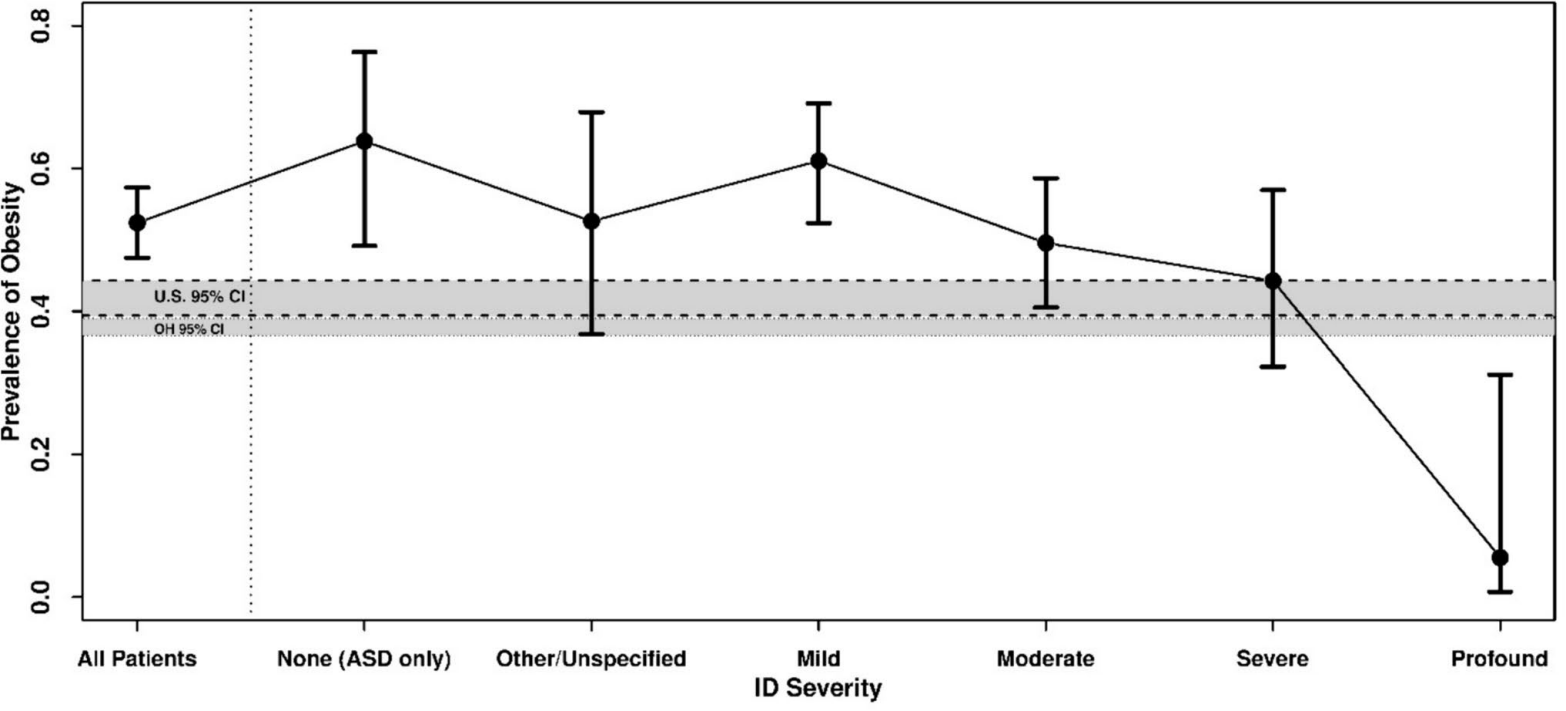
Prevalence (and 95% confidence intervals (CI)) of obesity (BMI ≥ 30 kg/m.^2^) among patients in the Access Ohio Telepsychiatry Project, overall and by ID severity; shaded regions correspond to the 95% CIs for obesity prevalence in the United States (Stierman et al., 2021) and Ohio (Centers for Disease Control and Prevention, 2021)

Sixty-four percent of this sample had ID only, 11.4% had ASD only, and 24.5% had both ID and ASD (Table 1). Most patients had mild (31.8%) or moderate (28.4%) ID. Only 14.8% of patients had severe ID and 4.4% had profound ID. The average age was about 41 years. Most patients were white (91%), and a majority were male (63.3%). Average BMI was 31.7 kg/m^2^ (obese class I). The average CNI was 3.14 on a scale of 1 to 5. Only 13.6% of patients had a known genetic disorder, with Trisomy 21 (2.7%) and Fragile X Syndrome (1.5%) being the most common. Respiratory, cardiovascular, and endocrine comorbidities were noticeably more prevalent among those in obese class III. Almost all (96.4%) patients had at least one comorbid psychiatric diagnosis, with the majority of patients actively treated with a second-generation antipsychotic (66.3%) or antidepressant (80.3%). In this sample, 78.4% (95% CI 74.0%, 82.2%) were overweight+ and 52.4% (95% CI 47.5%, 57.3%) were obese (Table 2). Obesity was most prevalent (63.8%) in patients with no ID (ASD only) and generally decreased with increasing ID severity (Table 3, Fig. 1).

**Table 1:** Patient characteristics by BMI category

Variable	All patients N = 412^a^	Not overweight N = 89 (21.6%)^a^	Over-weight N = 107 (26.0%)^a^	Obese N = 95 (23.1%)^a^	Obese class II N = 57 (13.8%)^a^	Obese class III N = 64 (15.5%)^a^
ID	365 (88.6%)	81 (91.0%)	98 (91.6%)	78 (82.1%)	48 (84.2%)	60 (93.8%)
Autism spectrum disorder (F84.0)	148 (35.9%)	35 (39.3%)	37 (34.6%)	41 (43.2%)	19 (33.3%)	16 (25.0%)
ID/ASD status						
ID only	264 (64.1%)	54 (60.7%)	70 (65.4%)	54 (56.8%)	38 (66.7%)	48 (75.0%)
ASD only	47 (11.4%)	8 (9.0%)	9 (8.4%)	17 (17.9%)	9 (15.8%)	4 (6.2%)
Both	101 (24.5%)	27 (30.3%)	28 (26.2%)	24 (25.3%)	10 (17.5%)	12 (18.8%)
ID severity						
None (ASD only)	47 (11.4%)	8 (9.0%)	9 (8.4%)	17 (17.9%)	9 (15.8%)	4 (6.2%)
Other/unspecified	38 (9.2%)	7 (7.9%)	11 (10.3%)	4 (4.2%)	8 (14.0%)	8 (12.5%)
Mild	131 (31.8%)	23 (25.8%)	28 (26.2%)	29 (30.5%)	17 (29.8%)	34 (53.1%)
Moderate	117 (28.4%)	24 (27.0%)	35 (32.7%)	25 (26.3%)	18 (31.6%)	15 (23.4%)
Severe	61 (14.8%)	17 (19.1%)	17 (15.9%)	20 (21.1%)	4 (7.0%)	3 (4.7%)
Profound	18 (4.4%)	10 (11.2%)	7 (6.5%)	0 (0.0%)	1 (1.8%)	0 (0.0%)
Age (years)	40.80 (14.48)	40.94 (15.09)	41.30 (15.18)	40.07 (14.02)	40.21 (15.13)	41.39 (12.74)
Body mass index (kg/m^2^)	31.66 (8.42)	21.67 (2.32)	27.46 (1.55)	32.48 (1.46)	37.49 (1.35)	46.13 (5.58)
Sex assigned at birth						
Female	151 (36.7%)	33 (37.1%)	31 (29.0%)	30 (31.6%)	21 (36.8%)	36 (56.2%)
Male	261 (63.3%)	56 (62.9%)	76 (71.0%)	65 (68.4%)	36 (63.2%)	28 (43.8%)
Minority status						
White	191 (91.0%)	39 (88.6%)	48 (96.0%)	48 (92.3%)	22 (84.6%)	34 (89.5%)
Minority	19 (9.0%)	5 (11.4%)	2 (4.0%)	4 (7.7%)	4 (15.4%)	4 (10.5%)
Unknown	202	45	57	43	31	26
Community need index	3.14 (0.78)	3.05 (0.81)	3.31 (0.73)	3.02 (0.78)	3.08 (0.78)	3.19 (0.82)
Unknown	4	0	0	1	2	1
Any genetic diagnosis	56 (13.6%)	12 (13.5%)	19 (17.8%)	8 (8.4%)	10 (17.5%)	7 (10.9%)
Trisomy 21	11 (2.7%)	2 (2.2%)	4 (3.7%)	1 (1.1%)	3 (5.3%)	1 (1.6%)
Fragile X syndrome	6 (1.5%)	0 (0.0%)	1 (0.9%)	3 (3.2%)	1 (1.8%)	1 (1.6%)
Other genetic condition	39 (9.5%)	10 (11.2%)	14 (13.1%)	4 (4.2%)	6 (10.5%)	5 (7.8%)
Any comorbidity diagnosis	403 (97.8%)	86 (96.6%)	104 (97.2%)	93 (97.9%)	57 (100.0%)	63 (98.4%)
Psychiatric disorder (excluding ID and ASD)	397 (96.4%)	85 (95.5%)	102 (95.3%)	91 (95.8%)	57 (100.0%)	62 (96.9%)
Neurological disorder	149 (36.2%)	42 (47.2%)	44 (41.1%)	27 (28.4%)	10 (17.5%)	26 (40.6%)
Epilepsy or seizure disorder	80 (19.4%)	27 (30.3%)	26 (24.3%)	16 (16.8%)	4 (7.0%)	7 (10.9%)
Respiratory disease	62 (15.0%)	12 (13.5%)	14 (13.1%)	10 (10.5%)	8 (14.0%)	18 (28.1%)
Musculoskeletal disorder	33 (8.0%)	5 (5.6%)	10 (9.3%)	9 (9.5%)	4 (7.0%)	5 (7.8%)
Digestive disorder	134 (32.5%)	39 (43.8%)	38 (35.5%)	25 (26.3%)	11 (19.3%)	21 (32.8%)
Endocrine disorder	149 (36.2%)	33 (37.1%)	33 (30.8%)	27 (28.4%)	21 (36.8%)	35 (54.7%)
Cardiovascular disease	101 (24.5%)	20 (22.5%)	19 (17.8%)	25 (26.3%)	12 (21.1%)	25 (39.1%)
Other medical comorbidities that do not fit into any of the specified categories	121 (29.4%)	31 (34.8%)	32 (29.9%)	27 (28.4%)	14 (24.6%)	17 (26.6%)
Any medication	407 (98.8%)	88 (98.9%)	104 (97.2%)	95 (100.0%)	56 (98.2%)	64 (100.0%)
Atypical antipsychotic	273 (66.3%)	66 (74.2%)	69 (64.5%)	60 (63.2%)	32 (56.1%)	46 (71.9%)
Typical antipsychotic	50 (12.1%)	5 (5.6%)	15 (14.0%)	14 (14.7%)	8 (14.0%)	8 (12.5%)
Antidepressant	331 (80.3%)	64 (71.9%)	82 (76.6%)	78 (82.1%)	51 (89.5%)	56 (87.5%)
Benzodiazepine	116 (28.2%)	28 (31.5%)	32 (29.9%)	26 (27.4%)	14 (24.6%)	16 (25.0%)
Stimulant	29 (7.0%)	4 (4.5%)	7 (6.5%)	7 (7.4%)	6 (10.5%)	5 (7.8%)
Beta blocker	30 (7.3%)	7 (7.9%)	7 (6.5%)	8 (8.4%)	5 (8.8%)	3 (4.7%)
Alpha blocker	11 (2.7%)	1 (1.1%)	1 (0.9%)	5 (5.3%)	3 (5.3%)	1 (1.6%)
Alpha agonist	86 (20.9%)	22 (24.7%)	20 (18.7%)	21 (22.1%)	13 (22.8%)	10 (15.6%)
Anti-epileptic	192 (46.6%)	39 (43.8%)	49 (45.8%)	43 (45.3%)	29 (50.9%)	32 (50.0%)
Anti-cholinergic	65 (15.8%)	18 (20.2%)	15 (14.0%)	14 (14.7%)	9 (15.8%)	9 (14.1%)
Other medications that do not fit in any of the specified categories	236 (57.3%)	54 (60.7%)	50 (46.7%)	59 (62.1%)	36 (63.2%)	37 (57.8%)

^a^n (%); Mean (SD)

**Table 2:** Prevalence of BMI categories

Prevalence of each level			Prevalence of at least each level	
Level		% (95% CI)	Level	% (95% CI)
Overweight	(25 ≤ BMI < 30)	26.0 (21.9, 30.5)	Overweight +	78.4 (74.0, 82.2)
Obese class I	(30 ≤ BMI < 35)	23.1 (19.1, 27.5)	Obese	52.4 (47.5, 57.3)
Obese class II	(35 ≤ BMI < 40)	13.8 (10.7, 17.6)	Obese Class II-III	29.4 (25.1, 34.1)
Obese class III	(BMI ≥ 40)	15.5 (12.2, 19.5)	Obese Class III	15.5 (12.2, 19.5)

**Table 3:** Prevalence of overweight + (BMI ≥ 25 kg/m^2^), obese (BMI ≥ 30 kg/m^2^), and obesity class II-III (BMI ≥ 35 kg/m^2^) by ID severity

ID severity	N	Overweight +	Obese	Obese class II–III
		% (95% CI)	% (95% CI)	% (95% CI)
None (ASD only)	47	83.0 (69.3, 91.3)	63.8 (49.1, 76.3)	27.7 (16.7, 42.2)
Other/unspecified	38	81.6 (65.9, 91.0)	52.6 (36.8, 67.9)	42.1 (27.5, 58.3)
Mild	131	82.4 (74.9, 88.1)	61.1 (52.4, 69.1)	38.9 (30.9, 47.6)
Moderate	117	79.5 (71.1, 85.9)	49.6 (40.5, 58.7)	28.2 (20.7, 37.1)
Severe	61	72.1 (59.5, 82.0)	44.3 (32.3, 57.0)	11.5 (5.5, 22.3)
Profound	18	44.4 (23.8, 67.2)	5.6 (0.8, 31.2)	5.6 (0.8, 31.2)

In secondary analyses, results for overweight+ and obesity were generally similar (Table 4). After adjusting for age, sex, and CNI, the odds of obesity were greatest in those with ASD alone, followed by those with ID alone. Those with ASD alone were observed to have over twice the odds of obesity as those with both ID and ASD (AOR 2.29, 95% CI 1.11, 4.87). However, overall, ID/ASD status was not significantly associated with obesity (p = .073) (Table 4). There was a statistically significant inverse relationship between ID severity and obesity (p < .001). Patients with other genetic conditions that do not fit into any of the specified categories (AOR 0.46, 95% CI 0.22, 0.92, p = .032), digestive (AOR 0.56, 95% CI 0.36, 0.87, p = .010), neurological (AOR 0.54, 95% CI 0.35, 0.81, p = .004), and seizure disorders (AOR 0.38, 95% CI 0.22, 0.63, p < .001) had significantly decreased odds of obesity. Patients with cardiovascular comorbidities (AOR 1.84, 95% CI 1.14, 3.00, p = .013) and those taking an antidepressant (AOR 2.03, 95% CI 1.23, 3.41, p = .006) had significantly increased odds of obesity (Table 4).

**Table 4:** Unadjusted and adjusted logistic regression results for overweight + and obese (adjusted models were adjusted for age, sex, and CNI; *OR* odds ratio, *AOR* adjusted OR)

Predictor	Level	Overweight + (BMI ≥ 25 kg/m^2^)				Obese (BMI ≥ 30 kg/m^2^)			
		Unadjusted		Adjusted		Unadjusted		Adjusted	
		OR (95% CI)	p	AOR (95% CI)	p	OR (95% CI)	p	AOR (95% CI)	p
ID/ASD status	Both *(reference)*		.314		.341		.108		.073
	ID only	1.42 (0.83, 2.40)	.198	1.40 (0.78, 2.51)	.256	1.35 (0.85, 2.14)	.201	1.37 (0.83, 2.28)	.223
	ASD only	1.78 (0.76, 4.53)	.199	1.75 (0.73, 4.57)	.225	**2.11 (1.04, 4.37)**	**.040**	**2.29 (1.11, 4.87)**	**.028**
ID severity	None (ASD only) *(reference)*		**.020**		**.024**		** < .001**		** < .001**
	Other/Unspecified	0.91 (0.29, 2.85)	.866	0.77 (0.23, 2.58)	.664	0.63 (0.26, 1.50)	.298	0.54 (0.21, 1.39)	.203
	Mild	0.96 (0.38, 2.26)	.934	0.90 (0.33, 2.26)	.828	0.89 (0.44, 1.76)	.738	0.72 (0.33, 1.52)	.391
	Moderate	0.79 (0.31, 1.86)	.610	0.72 (0.26, 1.82)	.499	0.56 (0.27, 1.11)	.100	0.47 (0.21, 1.01)	.055
	Severe	0.53 (0.20, 1.33)	.189	0.48 (0.17, 1.29)	.156	**0.45 (0.20, 0.97)**	**.045**	**0.40 (0.17, 0.91)**	**.031**
	Profound	**0.16 (0.05, 0.53)**	**.003**	**0.16 (0.04, 0.55)**	**.004**	**0.03 (0.00, 0.18)**	**.002**	**0.02 (0.00, 0.15)**	**.001**
Age		1.00 (0.98, 1.02)	.918	1.00 (0.98, 1.01)	.805	1.00 (0.98, 1.01)	.655	1.00 (0.98, 1.01)	.586
Sex	Male	1.02 (0.63, 1.66)	.925	1.01 (0.62, 1.65)	.956	0.72 (0.48, 1.08)	.109	0.72 (0.48, 1.09)	.119
CNI		1.21 (0.90, 1.63)	.213	1.21 (0.90, 1.64)	.209	0.84 (0.65, 1.08)	.180	0.85 (0.66, 1.09)	.211
Any genetic	Yes	1.01 (0.52, 2.09)	.973	1.03 (0.53, 2.15)	.923	0.70 (0.39, 1.23)	.211	0.67 (0.37, 1.18)	.167
Other genetic	Yes	0.75 (0.36, 1.68)	.460	0.74 (0.35, 1.68)	.449	**0.50 (0.24, 0.98)**	**.047**	**0.46 (0.22, 0.92)**	**.032**
Psych (excl. ID/ASD)	Yes	1.33 (0.36, 4.01)	.628	1.37 (0.36, 4.26)	.608	1.68 (0.60, 5.11)	.331	1.65 (0.57, 5.12)	.360
Neurological	Yes	**0.55 (0.34, 0.89)**	**.015**	**0.53 (0.33, 0.87)**	**.011**	**0.53 (0.35, 0.79)**	**.002**	**0.54 (0.35, 0.81)**	**.004**
Seizure	Yes	**0.45 (0.26, 0.78)**	**.004**	**0.43 (0.25, 0.74)**	**.002**	**0.39 (0.23, 0.64)**	** < .001**	**0.38 (0.22, 0.63)**	** < .001**
Respiratory	Yes	1.18 (0.61, 2.41)	.641	1.18 (0.61, 2.44)	.629	1.31 (0.76, 2.28)	.336	1.37 (0.79, 2.40)	.262
Musculoskeletal	Yes	1.59 (0.65, 4.81)	.352	1.57 (0.61, 4.85)	.388	1.10 (0.54, 2.27)	.800	1.17 (0.55, 2.52)	.686
Digestive	Yes	**0.53 (0.33, 0.87)**	**.011**	**0.52 (0.31, 0.86)**	**.010**	**0.55 (0.36, 0.84)**	**.005**	**0.56 (0.36, 0.87)**	**.010**
Endocrine	Yes	0.95 (0.59, 1.56)	.839	0.95 (0.57, 1.60)	.845	1.23 (0.82, 1.84)	.316	1.30 (0.85, 2.01)	.233
CVD	Yes	1.15 (0.67, 2.06)	.613	1.20 (0.68, 2.20)	.536	**1.62 (1.03, 2.58)**	**.039**	**1.84 (1.14, 3.00)**	**.013**
Other diagnosis	Yes	0.72 (0.44, 1.20)	.202	0.71 (0.42, 1.20)	.195	0.77 (0.51, 1.18)	.239	0.79 (0.50, 1.23)	.294
Atypical antipsychotic	Yes	0.62 (0.36, 1.04)	.077	0.60 (0.34, 1.00)	.056	0.80 (0.53, 1.20)	.285	0.80 (0.53, 1.22)	.298
Typical antipsychotic	Yes	**2.72 (1.14, 8.04)**	**.040**	2.56 (1.06, 7.61)	.057	1.42 (0.78, 2.63)	.254	1.53 (0.83, 2.88)	.175
Antidepressant	Yes	**1.86 (1.07, 3.19)**	**.025**	**1.98 (1.13, 3.43)**	**.015**	**2.04 (1.25, 3.39)**	**.005**	**2.03 (1.23, 3.41)**	**.006**
Benzodiazepine	Yes	0.82 (0.49, 1.37)	.434	0.78 (0.47, 1.32)	.343	0.79 (0.52, 1.22)	.291	0.78 (0.50, 1.20)	.258
Stimulant	Yes	1.78 (0.67, 6.18)	.295	2.07 (0.74, 7.41)	.207	1.53 (0.71, 3.42)	.284	1.44 (0.64, 3.35)	.388
Beta blocker	Yes	0.90 (0.39, 2.33)	.811	0.84 (0.36, 2.20)	.707	1.04 (0.49, 2.22)	.918	1.04 (0.48, 2.25)	.920
Alpha agonist	Yes	0.75 (0.44, 1.33)	.314	0.73 (0.41, 1.34)	.302	0.94 (0.58, 1.51)	.792	0.95 (0.57, 1.58)	.848
Anti-epileptic	Yes	1.15 (0.72, 1.86)	.553	1.12 (0.70, 1.81)	.646	1.14 (0.77, 1.68)	.509	1.17 (0.79, 1.73)	.442
Anti-cholinergic	Yes	0.67 (0.37, 1.25)	.196	0.63 (0.34, 1.18)	.134	0.86 (0.50, 1.46)	.574	0.91 (0.53, 1.56)	.727
Other medications	Yes	0.84 (0.52, 1.35)	.465	0.83 (0.51, 1.35)	.452	1.39 (0.94, 2.06)	.099	1.41 (0.94, 2.11)	.095

Bold values indicate the signifies p < 0.05

The ID severity × age interaction was not statistically significant for either overweight+ (p = .603) or obesity (p = .627). Therefore, we do not have sufficient evidence to reject the null hypothesis of no effect modification. Additionally, no clear patterns were evident in the ID severity AORs by age.

## Discussion

The results of the primary investigation support the hypothesis that the prevalence of obesity is higher in this patient sample (52.4%, with a lower confidence limit of 47.5%) than in the general population of the United States (41.9%) or in Ohio (37.8%) (Stierman et al., 2021; Centers for Disease Control and Prevention, 2021) (Fig. 1). Though a conclusion that ID and ASD are causally related to obesity cannot be drawn from this retrospective, correlational study, possible explanations are likely multifactorial, as is obesity itself. Compared to the general population, those with ID have varying degrees of ability and control in managing their diet and exercise, likely impacting weight over time. Furthermore, after the height of the COVID-19 pandemic in 2020, there was a shift to virtual work, school, and socializing, which may have influenced activity level for patients with ASD and/or ID.

The secondary, exploratory analysis revealed several interesting findings. First, there appeared to be an inverse relationship between ID severity and obesity prevalence. That is, those with less severe ID had greater odds of OW/OB. Secondly, the odds of obesity were greater in patients with a diagnosis of ASD only as opposed to patients with a diagnosis of ID only or ASD + ID co-occurring diagnoses.

The inverse relationship between ID severity and obesity prevalence suggests that physicians caring for those with less severe ID should have a *lower* threshold to screen for obesity and related sequelae compared to those with more severe ID. This finding is consistent with the results of existing literature, mainly cross-sectional, observational studies carried out in different clinical contexts. The composition and demographics of this study’s patient sample differed compared to other studies, for example one conducted in Ireland (Ryan et al., 2021), where patients enrolled were older than the patients seen through the Ohio Telepsychiatry Project. It is important to continue to explore different variables as potential modifiers to the relationship between ID and obesity while keeping the clinical sample composition in mind.

Lower severity of ID may be associated with more choices and less restrictions on diet, snacks, and activities. It is important to note that the sample in our study was taken from a telepsychiatry clinic, so all patients had some sort of psychiatric comorbidity. One study (Gast et al., 2018) exploring diet quality in people with and without ID found that those with milder ID had lower mean diet quality compared to those with severe to profound ID. These authors suggest that a change in eating habits could reduce disease burden. Results of our research, along with findings from Gast et al., support the implementation of increased programming and public health education regarding healthy diet in ID communities, focusing on those with mild to moderate severity.

Secondly, our study found that the odds of obesity were greatest among patients with ASD alone than in patients with ID alone or ID + ASD co-occurring diagnoses. However, it is important to note that the “ASD only” sample was only 11.4%. A cross-sectional, observational study of patients seen in the Kaiser Permanente system from 2008 to 2012 investigated the broader health status of adults with ASD (Croen et al., 2015). They found that Autistic adults had significantly increased rates of all major psychiatric disorders and nearly all medical conditions, including obesity, dyslipidemia, hypertension, and diabetes. They noted that social impairments and differences in sensory processing may prevent individuals with ASD from accessing preventative health care and reporting and localizing symptoms, possibly leading to delays in diagnosis and treatment of medical conditions. These impairments may also create barriers to maintaining a nutritious diet and participating in organized sports and regular physical activity. Considering the implications ASD often has on daily life may help explain our study’s finding of increased odds of obesity in patients with ASD.

Though it was hoped that this study would elucidate novel relationships between obesity, ID, and other comorbidities, few significant associations were found. It is noteworthy that in obesity class III, the proportions of patients with respiratory (28.1%), endocrine (54.7%), and cardiovascular (39.1%) comorbidities were much higher than in lower obesity classes, suggesting a stronger relationship between those and obesity. While this relationship is well-described in the literature, continued study of specific potential causes—for example, medications or access to care—would likely improve ID care.

These findings are important because they can guide clinical practice. Providers and IDD practitioners can and should expand their scope of practice to monitor for obesity in their high-risk populations, including those with severe mental illness and educate them about the risks of obesity (Chwastiak & Tek, 2014). This can be an opportune moment to spearhead multidisciplinary treatment plans including behavioral weight loss interventions, specialist referral, pharmacotherapy, or medication reconciliation to reach goals. Also, caregivers and staff (home health care, school aids, etc.) that work with these patients more closely can implement timely changes or modifications to diet and exercise. These members of the care team spend more quality time with patients and are often more in tune with each individual patient’s patterns and routines. Patients may be offered or independently consume familiar yet unhealthy foods to manage emotional dysregulation. This practice may help with short-term emotional regulation but can create a long-term issue of overweight/obesity. Future studies may consider exploring how to manage diet in a way that is efficacious, ethical, and specific to the needs and cognition of patients in this population while preserving their autonomy.

There were several limitations to this study. BMI’s utility as an indicator or predictor of health has recently been called into question, given its roots as an observational measure to assess weight/height proportions in only white men (American Medical Association, 2023). In addition, Ranjan et al. (2018) notes that individuals with IDD often display unique anthropometry compared to individuals without disability, calling into question how accurately BMI can be used as a measure of health status. Nevertheless, BMI continues to be a widely accepted marker for measuring overweight/obesity, especially given its accessibility and ease (Gutin, 2018). As this study was a retrospective chart review, obtaining BMI was more feasible compared to using tools such as waist-to-hip ratio.

Another limitation was a lack of a more demographically diverse sample. Race/ethnicity was not available for almost half of the patients and, among those for whom it was known, almost all were white (91.0%). This prevented a robust investigation of the potential relationship between obesity and race in patients with ID/ASD. Therefore, this sample cannot necessarily be compared to the general population of those with ID. This has important implications in the care of adults with ID from different cultures or communities with various viewpoints on food, value of weight, and stigma surrounding ID. Additionally, most patients in our study with ID fell in the mild to moderate ID severity categories, therefore each category of ID severity was not equally well-represented to better elucidate any possible correlates.

Retrospective data accuracy depends on the accuracy of documentation in the medical record. Because this patient sample is engaging in telepsychiatry, measurement of weight is obtained either independently or with the help of caretakers. Because of this, there is likely some degree of variability weight measurements given the variation in scales and difference in caretaker method. Looking at patients who were active for 1 year may have also limited the data, as this automatically excludes those patients that may have been lost to follow-up. Living situation (independent, group home, etc.) has been thought to influence OW/OB in these populations; yet such data was not readily extractible during the data abstraction phase.

These limitations suggest additional avenues for future study—a more demographically diverse (in age, sex, race, ID severity) patient sample as well as better measures of obesity, may further clarify the relationship between ID/ASD and obesity. Additionally, it may prove useful to further study the relationship between living environment (independent/with family versus group home versus residential care center) and obesity in patients with ID/ASD. Additional analysis into the relationship between ID/ASD and obesity can benefit those outside of the United States as well. The WHO notes that that while obesity was considered a high-income country problem in the past, obesity is now on the rise in low- and middle-income countries. It is imperative when designing early screening and intervention programs to account for the difference in resources available to ASD/ID populations in these countries compared to the plethora of resources available to those in the United States.

## Conclusion

These trends outlined in Access Ohio Telepsychiatry Project serving many rural and underserved communities, may be extrapolated to other communities with varying intellectual and developmentally abled patients. The inverse relationship between overweight/obesity prevalence and ID severity may have important implications to patients. To avoid speculation of causation, further research is warranted to examine caregiver involvement, patient independence, patient access to dietetics, occupational therapy, physical therapy, and other healthcare adjacent disciplines that would influence one’s diet and mobility. Providers also have a unique opportunity to understand and treat the interface of complex behavior and insight within IDD and its intersections with overweight/obesity within a patient’s interdisciplinary health care team.

The higher prevalence of overweight/obesity in ASD diagnoses alone compared to co-occurring ASD/ID or ID alone should signal to practitioners and families the increased risk for metabolic syndrome and perhaps even cardiovascular sequelae when a diagnosis of ASD is made. This certainly has important implications when discussing preventative medicine within ASD populations in the primary care setting, as parents, caregivers, and clinicians alike can be empowered to watch for obesity early in childhood.

All patients with ID, ASD, or ID + ASD would further benefit from policymakers and developmental disability agencies advocating and implementing programming to increase accessibility to healthy foods, to public activity spaces, and to dietetics specialists. Public health strides to prevent obesity early in childhood and young adolescence that are specific and feasible to these populations are imperative to the long-term health of these individuals.

## Data Availability

Due to the highly specific nature of this data, the privacy of research subjects could be compromised and protective measures such as de-identification would be insufficient. Therefore, we do not plan to share the data.
